# Co-inoculation effect of *Rhizobium* and *Achillea millefolium* L. oil extracts on growth of common bean (*Phaseolus vulgaris* L.) and soil microbial-chemical properties

**DOI:** 10.1038/s41598-019-51587-x

**Published:** 2019-10-23

**Authors:** Veysel Turan, Peter Schröder, Serdar Bilen, Heribert Insam, Marina Fernández-Delgado Juárez

**Affiliations:** 1 Institute of Microbiology, University of Innsbruck, Innsbruck, Austria; 2 Department of Soil Science and Plant Nutrition, Faculty of Agriculture, Bingöl University, Bingöl, Turkey; 3 Department of Microbe-Plant Interactions, Helmholtz Zentrum München, German Research Center for Environmental Health GmbH Neuherberg, Oberschleißheim, Germany; 4 Department of Soil Science and Plant Nutrition, Faculty of Agriculture, Atatürk University, Erzurum, Turkey

**Keywords:** Plant sciences, Environmental sciences

## Abstract

Essential oils (EO) of several plant species have the potential to combat plant and fungal diseases. However, the effects of *Achillea millefolium* EO on the development of common bean (*Phaseolus vulgaris* L.), is still unknown. Moreover, its effect on N_2_-fixing bacteria, and in general on soil properties has not been studied yet. A greenhouse trial was set up to evaluate both the influence that *Achillea millefolium* EO and the inoculation with three different *Rhizobium* strains have on the bean plant and on the chemical and microbiological properties of an agriculturally used Cambisol. Non-inoculated pots were used as control. Our findings showed a decrease in bacterial colony forming units due to EO application and an increase following the *Rhizobium* inoculation compared to the control. The EO application decreased soil basal respiration and activities of dehydrogenase, urease, β-glucosidase and acid phosphatase. Such effects were stronger with higher oil concentrations. Moreover, the treatments combining *Rhizobium* inoculation with EO showed a positive effect on nodulation and plant height. Overall, the combined application of *Achillea millefolium* EO and rhizobia works as an efficient biocide that could be applied in organic agriculture without hampering the activity of nodule-forming N-fixing bacteria and the development of common bean.

## Introduction

It is expected that the world population will reach 9.8 billion people by 2050, and 11.2 billion in 2100 according to the United Nations^1^, and thus global agriculture will be intensified to feed the increasing population. Enhancing the productivity of agricultural systems while substantially reducing environmental impact is a worldwide responsibility in order to ensure food security for future generations.

Common bean (*Phaseolus vulgaris* L.) is one of the most important legumes in the world^2^. In areas of Eastern Africa and Latin America, this plant contributes to 65% of the total protein consumed and 32% of the energy intake^3^. Moreover, it has been estimated that approximately 26 million metric tons of beans were produced worldwide in 2016^4^, being a crucial crop in developing countries^5^. In developing areas, a sustainable practice to enhance common bean production consists in their inoculation with symbiotic and associative bacteria to enhance plant growth^6^.

Plant oils have been used for thousands of years for several purposes^7^ and are in ever increasing demand nowadays as an alternative medicine. Previous scientific studies have related essential oil compounds to their medical effectivity^8^. Another use for plant-essential oils is in the food industry due to their antibacterial, antifungal, antiviral, and antioxidant properties^9^. However, in agriculture, their application is connected to plant protection^10^ and for their further use as eco-friendly pesticides^11–13^. The essential oils of *Achillea* species have been the subject of several studies in which their therapeutic, cosmetic, and fragrant properties have been demonstrated. Işcan *et al*.^14^ studied the antimicrobial properties of several plants of the *Achillea* genus and found that the *Achillea aleppica* subsp. *aleppica* had moderate antimicrobial activity, similar to *Achillea millefolium*. Along these lines, Stojanovic *et al*.^15^ evaluated the *in vitro* antimicrobial effect of four different *Achillea* species confirming the antibacterial and fungicidal effect of *Achillea millefolium*. Despite the proven effect of *Achillea* essential oils in the medical field, their potential application in agriculture has not been studied yet, potentially opening a promising door for future applications, especially in biologically and dynamically managed agricultural fields.

It is widely known that diazotrophic bacteria, among them rhizobia, promote biological nitrogen (N) fixation resulting in better plant development^16,17^. Moreover, previous studies dealing with the effect of *Rhizobium* inoculation on plants have focused on soil and soil mineral N content^18^, nodulation^19^, nitrogen fixation^20^, plant height^21^ and plant growth^22^. All of these parameters are crucial to assess if the soil-microbe-plant interaction shall lead to enhanced plant performance.

The use of bacteria from the genus *Rhizobium* has been shown to be an environment-friendly alternative to mineral nitrogen fertilizers, due to their positive effects on plant nodulation, biological N-fixation and consequently N-availability. Although the fixed nitrogen is not readily available to the plant, inoculation ensures long-term N-availability in soil, thus, being an ecologically viable option for amelioration of agricultural soils^23^. In line with this, inoculation of *Rhizobium sp*. BARIRGm901 in gray terrace soils led to higher nitrogen fixation and yield of soybean (*Glycine max*)^24^. Moreover, *Rhizobium* inoculation does not only affect the plant performance but also the soil microbiota; indeed, Fall *et al*.^25^ found that *Rhizobium* inoculation on a Gum Arabic production area with natural trees of *Senegalia senegal* (L.) Britton, (synonym: *Acacia senegal*) led to an increase in soil microbial biomass and respiration as well as mineral nitrogen content; and positive effect on soil microbial functional diversity was also observed^25^. Numerous studies have examined the influence of plant essential oils with regard to plant protection as biological control agents, either alone or in combination with alternative fungicides. However, there is still scarce information on how soil productivity and plant growth are affected by the application of essential oils alone or in combination with *Rhizobium*, and if these affect the microbiota in an antagonistic or synergistic way.

The aims of the present study were to investigate the effects of *Achillea millefolium* essential oil and inoculation with different *Rhizobium* strains on soil biological properties as well as on the growth of bean (*Phaseolus vulgaris* L.) and its nutrient content. For this purpose, we determined the effects on soil enzyme activities, soil microbial biomass and respiration, plant biomass and plant nutrient content (N), together with soil nutrient content (e.g. mineral N) and plant root nodulation.

## Material and Methods

### Experimental setup

#### Soil sampling

In the present study, a factorial greenhouse pot experiment was set up in July, 2013 in the Helmholtz Zentrum München - German Research Center for Environmental Health (Neuherberg, Germany). The soil used was collected on a research farm in the municipality of Scheyern (Germany, 48°29′54.1″N 11°26′21.3″E). It was classified as Cambisol^26^, and its main physiochemical properties are shown in Table 1. After the sampling, the soil was air-dried, sieved (Ø < 2 mm), and sterilized by autoclaving three consecutive times at 121 °C for 30 min, prior to its distribution in sterile pots. After autoclaving, one kg sterile soil was placed in sterile pots (20 cm in diameter and 18 cm in height).

**Table 1:** Physiochemical properties of the soil used in this study.

Parameters	Units	Values
Sand	%	21.7 ± 1.0
Silt	%	46.4 ± 0.9
Clay	%	31.9 ± 1.9
Organic Matter	%	3.28 ± 0.1
CaCO_3_	%	0.47 ± 0.1
pH	—	6.50 ± 0.1
EC^a^	dS m^−1^	0.52 ± 0.1
CEC^b^	cmol_c_ kg^−1^	35.7 ± 0.8
Phosphorus	mg kg^−1^	9.78 ± 0.1
Total Nitrogen	mg kg^−1^	250.3 ± 9.5
N-Ammonium	mg kg^−1^	19.1 ± 1.2
N-Nitrate	mg kg^−1^	29.9 ± 0.6

Values are given on a dry weight basis as an average of n = 3 ± standard deviation.

^a^Electrical conductivity.

^b^Cation exchange capacity.

#### Essential plant oil preparation

Leaves of *Achillea millefolium* plants were collected from the research campus at Helmholtz Zentrum München - for Environmental Health (Neuherberg, Germany) in June 2013. Freshly collected leaves were shade-dried at room temperature (22 ± 1 °C) for 10–15 days. Afterwards, the dried leaves were ground into a fine powder with a pestle and mortar. 50 gr of the sample was subjected to hydro distillation for 3 h in 500 mL distilled water using a Clevenger type apparatus for 4 h in order to produce essential oil. After distillation, the EO concentration was determined by gas chromatography-mass spectrometry (GC-MS). Concentrated EO (1500 ppm) were then diluted using distilled water to the following concentrations: 0 ppm: 0 ml EO 100 ml^−1^, 100 ppm: 6.66 EO 100 ml^−1^, and 1000 ppm: 66.6 EO ml 100 ml^−1^.

#### Rhizobium inoculum preparations

*Rhizobium leguminosarum biovar phaseoli* cultures were obtained from the laboratory stock culture collection and prepared by sub-culturing in three mL of YMA (yeast extract–mannitol agar) and incubated at 28 °C for 5 days. Rhizobial inocula *(Rhizobium leguminosarum biovar phaseoli F7*, *Rhizobium leguminosarum biovar phaseoli F83*, *Rhizobium leguminosarum biovar phaseoli Ciat 899)* were incubated at 30 °C for three days in YMA, and were posterior used as material for inoculation in this experiment^27^. In order to have equal numbers of bacteria as inoculation material, absorbance values at 540 nm wavelength were measured spectrophotometrically. To achieve an equal bacterial density, the lowest number of bacteria after 24 h was taken as a baseline. Where appropriate, bacterial suspensions were diluted to the same absorbance value with sterile physiological saline solution (PSS)^27^.

### Greenhouse set up

A total of twelve treatments resulted from combining three different *Achillea millefolium* essential oil doses (E0 (control), E1 (100 mg kg^−1^) and E2 (1000 mg kg^−1^)) per kg of pot soils with three different *Rhizobium* inocula and a non-inoculated control (R0: No inoculation; R1: *Rhizobium leguminosarum biovar phaseoli* F7; R2: *Rhizobium leguminosarum biovar phaseoli* F83; R3: *Rhizobium leguminosarum biovar phaseoli* Ciat 889). All treatments were done in triplicate, with a total of 36 pots.

- R0 E0 No inoculation/No essential oil
- R0 E1 No inoculation/essential oil (100 mg kg^−1^)
- R0 E2 No inoculation/essential oil (1000 mg kg^−1^)
- R1 E0 *Rhizobium leguminosarum biovar phaseoli* F7/No essential oil
- R1 E1 *Rhizobium leguminosarum biovar phaseoli* F7/essential oil (100 mg kg^−1^)
- R1 E2 *Rhizobium leguminosarum biovar phaseoli* F7/essential oil (1000 mg kg^−1^)
- R2 E0 *Rhizobium leguminosarum biovar phaseoli* F83/No essential oil
- R2 E1 *Rhizobium leguminosarum biovar phaseoli* F83/essential oil (100 mg kg^−1^)
- R2 E2 *Rhizobium leguminosarum biovar phaseoli* F83/essential oil (1000 mg kg^−1^)
- R3 E0 *Rhizobium leguminosarum biovar phaseoli* Ciat 889/No essential oil
- R3 E1 *Rhizobium leguminosarum biovar phaseoli* Ciat 889/essential oil (100 mg kg^−1^)
- R3 E2 *Rhizobium leguminosarum biovar phaseoli* Ciat 889/essential oil (1000 mg kg^−1^)

*Phaseolus vulgarıs* L seeds were surface-sterilized in 5% H_2_O_2_ for 15 minutes and rinsed 5 times with sterile distilled water. After the soils were potted, each pot was planted with three sterile seeds. After seedling, the two seedlings showing the weakest growth were removed 7 days after planting. The essential oils were diluted (0, 100 and 1000 ppm) with 10 ml ethyl alcohol and were slowly surface-sprayed. Seven days after planting, each seedling was inoculated with one mL of log phase bacterial culture with the help of a sterile syringe on the soil surface in the root area. During the 45-day incubation period, soil moisture content was kept at field capacity. The pots were irrigated with water containing bacterial culture suspensions (diluted to 1 × 10^9^ cells in each pot) to 55–60% of the soil water-holding capacity. Soils were maintained at this moisture level by watering them to weight every 2–3 days. After the incubation period, plants were removed from the pots and soil samples were collected and kept at 4 °C prior to analysis.

### Soil physico-chemical analyses

Soil electrical conductivity (EC) and pH were determined in 10 g of soil in combination with 25 mL A.d. at a ratio of 1:2.5. Soil texture was analyzed according to Bouyoucos^28^. Soil cation exchange capacity (CEC) and organic matter (OM) were analyzed as described in Sumner and Miller^29^ and Nelson and Sommers^30^ respectively. Soil inorganic nitrogen (N-NH_4_^+^ and N-NO_3_^−^) was determined in 2 M KCl extracts according to Bremmer and Keeney^31^. To determine extractable inorganic P, soil samples were extracted with 0.5 M NaHCO_3_ (pH 8.5) for 30 min, and P was measured following the method proposed by Olsen and Sommers^32^.

### Soil biological analyses

#### Soil bacterial and fungal abundance

Bacterial and fungal abundance in the soil samples were assessed by determining colony forming units (CFU). One g of soil was extracted in 9 mL phosphate buffered saline (PBS) in a rotation shaker for 30 min. Samples were diluted 10^−6^ and 10^−7^, and 100 μL of each sample was then plated on soil extract medium and dextrose-peptone agar for bacteria and fungi, respectively. Bacterial plates were incubated at 30 °C, and fungal ones at 25 °C for a total of seven days^33^. In the case of bacterial colonies, the average CFUs per gram of oven-dried equivalent (ODE) of field-moist soil was calculated by using an automated colony counter^34,35^. Fungal colonies growing in agar were observed under a dissecting microscope. They were then marked and recorded in the surface agar. Total culturable fungi were calculated by viable fungal spore g^−1^ of oven dry soil^36^. It has to be addressed that this culture‐dependent method is biased because it generally underestimates the total cell number by a factor of 10–100^37^.

#### Soil microbial activity

Soil basal respiration (BR) was determined according to Islam and Weil^38^. Briefly, the CO_2_ released from 20–25 g moist soil sample previously incubated at 25 ± 1 °C for 24 h was then trapped in 20 mL of 0.5 M NaOH solution. Afterwards, BaCl_2_ was added to precipitate the CO_2_ as BaCO_3_, followed by a titration with 0.5 M HCl.

#### Soil enzyme activities

Five soil enzyme activities were measured: acid phosphatase (AcdP) and alkaline phosphatase (AlkP) activities were assessed by using pNPP (para-Nitrophenyl phosphate) as substrate and expressed as µg pNP g DM^−1^ h^−1^ following the method of Tabatabai^39^. Soil urease (UE) activity was determined using the steam distillation methods as described by Tabatabai and Bremner^40^ using urea as substrate and expressed as µg NH_4_-N g DM^−1^ 2 h^−1^. β-glucosidase activity (β-Glu) was assayed using β-PNPGLU (p-Nitrophenyl-β-D-glucopyranoside) as substrate and expressed as µg PNP g DM^−1^ h^−1^ ^39^. Dehydrogenase (DH) activity was determined with the TTC (triphenyl tetrazolium chloride) method^39^ and expressed as µg TPF g DM^−1^ 24 h^−1^.

### Plant analyses

The growth response (plant height), total plant biomass, nodule number, and plant total N-content were measured in this experiment, as these parameters are well-known to show a quick response when plants are inoculated with nodule-forming N-fixing bacteria. Both the aboveground (leaf and stem) and root biomass were collected separately from the experimental pots and washed. Roots were oven-dried at 65 °C for 72 h, while leaves and stems were dried for 48 h to determine their dry matter content. After the plant harvest, the numbers of nodules per plant were determined in the roots. Total plant nitrogen contents were determined by the Kjeldahl method after salicylic-sulfuric acid digestion^41^.

### Statistical analyses

Statistical analyses were performed using the SPSS 23 Software (2015, IBM Corp. Armonk, NY, USA). Data were analyzed using a two-way analysis of variance (ANOVA). When significant differences were obtained, an analysis with Tukey’s HSD (honestly significant difference) comparison procedure was further performed as a post hoc test. Prior to analysis, data were tested for normality and transformed to meet this condition when necessary. When data did not meet the normality assumption, they were subjected to non-parametric tests for several independent samples (Kruskal–Wallis test) and pair-wise comparisons were performed using the non-parametric Friedman test.

## Results

### Influence of essential oil and Rhizobium inoculation on soil chemical properties

The inoculation of soil with different *Rhizobium* strains and the application of different doses of *Achillea millefolium* essential oil as well as their interaction did not induce significant effects (*p* > 0.05) on different soil N-pools (nitrate, ammonium and total N) (Tables 2 and 4). Despite there were no significant effects of the different treatments on the soil ammonium content, soils inoculated with R2 and R3 had higher ammonium contents than the remaining soils. A similar trend was recorded for the soil nitrate content for which no significant differences were recorded among treatments (Tables 2 and 4). In particular, for soils inoculated with *Rhizobium* species (R1, R2, and R3) the application of E1 induced an increase in the nitrate content, while no such increase was observed for E2.

**Table 2:** Effects of different rates of essential oil (E0: No essential oil; E1: essential oil (100 mg kg^−1^), E2: essential oil (1000 mg kg^−1^)) and different *Rhizobium* inocula (R0: No inoculation, R1: *Rhizobium leguminosarum biovar phaseoli F7*, R2: *Rhizobium leguminosarum biovar phaseoli F83*, R3: *Rhizobium leguminosarum biovar phaseoli Ciat899)* on soil chemical and microbial properties.

Treatments		Parameters					
Inoculation	Essential Oil	Total N (%)	NO_3_^−^ (µg N g^−1^)	NH_4_^±^ (µg N g^−1^)	Bacterial abundance (CFU g^−1^)	Fungal abundance (CFU g^−1^)	Basal respiration (µg C g^−1^ h^−1^)
No inoculation (R0)	E0	0.19 ± 0.01	55.1 ± 18.0	15.3 ± 0.6	3.34 · 10^7^ ± 8.91 · 10^6^ ab	5.50 · 10^6^ ± 0.12 · 10^6^ a	24.9 ± 0.4 a
	E1	0.20 ± 0.01	43.3 ± 6.9	12.8 ± 3.3	3.78 · 10^7^ ± 7.55 · 10^6^ a	5.53 · 10^6^ ± 3.73 · 10^6^ a	17.0 ± 3.0 b
	E2	0.21 ± 0.01	34.1 ± 6.3	17.0 ± 11.8	1.35 · 10^7^ ± 1.38 · 10^6^ b	3.16 · 10^6^ ± 1.38 · 10^6^ b	13.7 ± 2.8 b
*R1*	E0	0.20 ± 0.02	46.8 ± 20.5	16.6 ± 6.4	2.65 · 10^7^ ± 2.38 · 10^6^ a	6.06 · 10^6^ ± 0.21 · 10^6^ b	26.5 ± 2.8 a
	E1	0.18 ± 0.06	67.6 ± 29.8	17.5 ± 4.2	0.74 · 10^7^ ± 4.17 · 10^6^ b	9.73 · 10^6^ ± 4.17 · 10^6^ a	17.2 ± 3.4 b
	E2	0.18 ± 0.03	60.1 ± 26.1	12.7 ± 2.7	0.82 · 10^7^ ± 0.71 · 10^6^ b	4.16 · 10^6^ ± 0.71 · 10^6^ c	13.3 ± 5.4 b
*R2*	E0	0.20 ± 0.01	42.0 ± 17.5	21.0 ± 3.6	1.43 · 10^7^ ± 9.37 · 10^6^ a	3.73 · 10^6^ ± 1.37 · 10^6^ c	15.5 ± 4.8 a
	E1	0.19 ± 0.02	55.6 ± 23.3	14.6 ± 1.5	0.69 · 10^7^ ± 1.82 · 10^6^ c	4.63 · 10^6^ ± 1.82 · 10^6^ b	17.3 ± 1.6 a
	E2	0.20 ± 0.01	43.9 ± 9.4	11.5 ± 0.8	1.32 · 10^7^ ± 0.53 · 10^6^ b	16.1 · 10^6^ ± 2.20 · 10^6^ a	15.4 ± 1.5 a
*R3*	E0	0.19 ± 0.03	48.9 ± 4.0	15.7 ± 2.4	7.60 · 10^7^ ± 1.83 · 10^6^ a	7.60 · 10^6^ ± 3.16 · 10^6^ a	14.5 ± 2.8 b
	E1	0.20 ± 0.02	52.1 ± 8.5	24.3 ± 11.6	2.65 · 10^7^ ± 2.40 · 10^6^ b	2.65 · 10^6^ ± 1.08 · 10^6^ c	13.5 ± 1.45 c
	E2	0.22 ± 0.02	34.3 ± 7.1	15.7 ± 1.2	1.39 · 10^7^ ± 2.19 · 10^6^ c	5.90 · 10^6^ ± 3.67 · 10^6^ b	23.7 ± 7.2 a

Different letters indicate significant differences followed by Tukey post- hoc test considering essential oil application for each inoculation treatment and interaction. Values are given as an average for n = 3 on a dry weight basis ± standard deviation.

**Table 3:** Effects of different rates of essential oil (E0: No essential oil; E1: essential oil (100 mg kg^−1^), E2: essential oil (1000 mg kg^−1^)) and different *Rhizobium* inocula (R0: No Inoculation, R1: *Rhizobium leguminosarum biovar phaseoli F7*, R2: *Rhizobium leguminosarum biovar phaseoli F83*, R3: *Rhizobium leguminosarum biovar phaseoli Ciat899*) on nodules, plant height, total plant biomass and plant N content.

Treatments		Plant parameters of *Phaseolus vulgaris L*.			
Inoculation	Essential Oil	Nodules	Plant height (cm)	Total plant biomass (g DW)	Plant N content (g kg^−1^)
No inoculation (R0)	E0	0 ± 0.0 a	35.3 ± 1.5	2.96 ± 0.6	1.70 ± 0.5
	E1	0 ± 0.0 a	34.3 ± 1.5	2.87 ± 0.4	1.80 ± 1.1
	E2	0 ± 0.0 a	27.0 ± 2.0	2.78 ± 0.7	1.98 ± 0.5
*R1*	E0	22.7 ± 6.6 a	36.3 ± 9.5 a	3.31 ± 0.8	2.24 ± 0.8
	E1	36.7 ± 9.0 b	28.3 ± 1.5 b	3.22 ± 0.9	2.42 ± 0.5
	E2	29.0 ± 1.7 ab	39.0 ± 9.2 a	3.39 ± 0.7	2.49 ± 1.4
*R2*	E0	48.3 ± 2.8 a	31.0 ± 10.5a	3.81 ± 0.8	1.66 ± 0.8
	E1	40.7 ± 9.3 b	26.0 ± 3.6 b	3.63 ± 0.8	2.17 ± 0.9
	E2	44.0 ± 3.6 ab	35.0 ± 7.5 a	3.49 ± 1.7	2.69 ± 1.4
*R3*	E0	46.3 ± 2.9 a	48.0 ± 1.0 a	3.94 ± 1.0	2.58 ± 1.0
	E1	54.0 ± 3.0 b	42.0 ± 6.2 b	3.89 ± 0.9	2.45 ± 1.3
	E2	51.0 ± 1.00 ab	47.0 ± 2.6 a	3.54 ± 0.9	3.05 ± 1.7

Different letters indicate significant differences followed by Tukey post- hoc test considering essential oil application for each inoculation treatment. Values are given as an average for n = 3 on a dry weight basis ± standard deviation.

**Table 4:** Two-way analysis of variance (ANOVA) for the soil chemical and microbiological parameters of the soil treated with rhizobial inocula and essential oils as source of variance.

Parameters	Inoculation treatment		Essential oil treatment		Inoculation × Essential oil	
	*F*	*p*	*F*	*p*	*F*	*p*
Total N	n.s.	n.s.	n.s.	n.s.	n.s.	n.s.
NH_4_^+^	n.s.	n.s.	n.s.	n.s.	n.s.	n.s.
NO_3_^−^	n.s.	n.s.	n.s.	n.s.	n.s.	n.s.
Bacterial abundance	27.8	*p* < 0.001	26.6	*p* < 0.001	7.74	*p* < 0.001
Fungal abundance	n.s.	n.s.	n.s.	n.s.	0.28	*p* < 0.001
Basal respiration	n.s.	n.s.	4.90	*p* < 0.05	6.93	*p* < 0.001

n.s.: Not significant.

### Influence of essential oil and Rhizobium inoculation on soil biological properties

The application of essential oil doses to the soils significantly decreased bacterial abundance (Tables 2 and 4; *p* < 0.001), as did the inoculation with different *Rhizobium* strains (*p* < 0.001). Moreover, a significant interaction between the application of essential oil and *Rhizobium* strains (*p* < 0.001) was also found (Table 4). The bacterial abundance increased for R3 strain, but not for R1 and R2 strains, compared to the control pots where no essential oil was added (E0). The addition of essential oils to the soils induced a significant decrease in bacterial CFUs. When oil at a concentration of 100 ppm was added (E1), a reduction of approximately 30% in bacterial CFUs was observed in all the inoculation treatments (R1, R2, R3), but not for the non-inoculated one (Table 2). The application of a dose 10 times higher (E2) only induce a further decrease for R2 and R3 treatments. Contrarily to what was observed for bacterial CFUs, only the interaction between the application of essential oil and *Rhizobium* strains (*p* < 0.001) was found to affect soil fungal CFUs (Table 4). Each combination of oil treatments x Inoculation affected the soil fungal abundance differently (Table 2). For the control soils, only high oil doses of 1000 ppm (E2) induced a decrease in the fungal abundance. In the case of R1 and R2 treatments, less concentrated oil application (E1) enhanced the fungal CFUs, while higher concentrations (E2) significantly decreased the number of culturable fungi. Contrarily, a sharp decrease in the fungal CFUs was observed for soil inoculated with the strain R3, ranging from 7.6·10^6^ to 2.65·10^6^, when applying a lower dose of essential oil (E1). A lower reduction was found for higher oil concentrations (E2).

As described above for the microbial CFUs, BR was negatively affected by the application of essential oils (*p* < 0.05). In general, the application of the oil extracts decreased of soil basal respiration (Table 2). A significant interaction between both, EO application and inoculation, was also observed (*p* < 0.001; Table 4). Both oil doses, E1 and E2, reduced BR, from 24.9 to 17.0 and 13.7 mg C kg^−1^ h^−1^, respectively. A decrease from 26.5 to 17.2 and 13.2 mg C kg^−1^ h^−1^ was observed for non-inoculated soils and R1 treatment, respectively. In contrast, when soil was inoculated with R3 BR increased up to 23.7 mg C kg^−1^ h^−1^ with regard to the higher oil doses (E2: 1000 ppm) (Table 4).

Regarding the effects of *Achillea millefolium L*. oil extracts on the activity of the different soil enzyme activities, no effects were observed, except for β-Glu (*p* < 0.05) for which a reduction was detected compared to the control.

**Figure 1:**
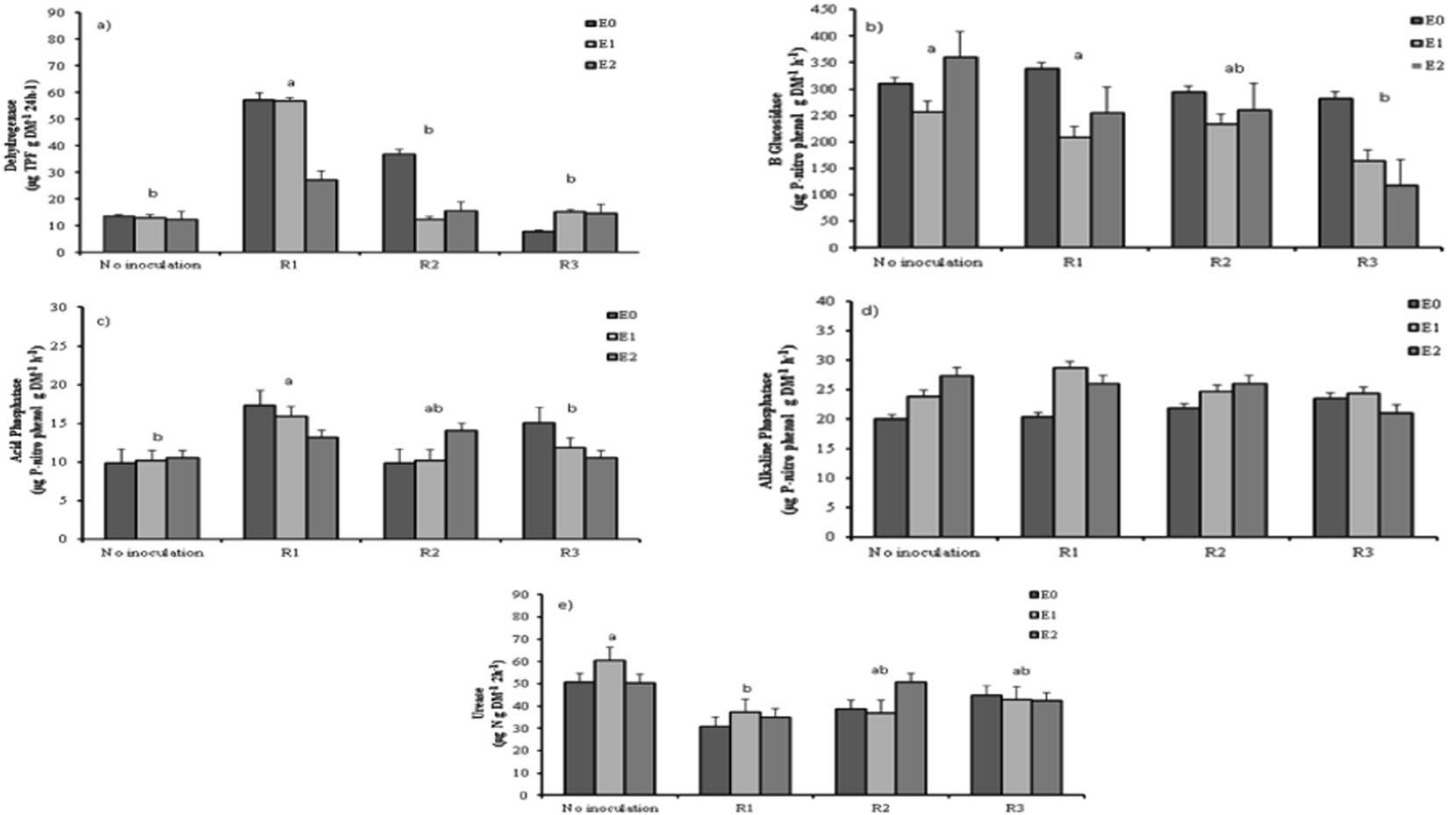
Effects of *Rhizobium* inoculation on (**a**) dehydrogenase enzyme activity, (**b**) β-Glucosidase enzyme activity, (**c**) acid phosphatase enzyme activity, (**d**) acid phosphatase enzyme activity, and (**e**) urease enzyme activity of soils amended with essential oil (E0: No essential oil; E1: essential oil (100 mg kg^−1^), E2: essential oil (1000 mg kg^−1^) and *Rhizobium* inoculation (R1: *Rhizobium leguminosarum biovar phaseoli* F7; R2: *Rhizobium leguminosarum biovar phaseoli* F83; R3: *Rhizobium leguminosarum biovar phaseoli* Ciat 889). Values are means ± SE. Different letters indicate significant differences at p < 0.001. Post doc comparisons have been done between the different inoculation treatments.

Four of the studied enzymes, that is DH, β-Glu, AcdP and urease (UE) showed a significant decline in their activity (*p < *0.001, for all enzymes), when soil was inoculated with the different *Rhizobium* strains (Fig. 1). Interestingly, neither the application of oils nor the *Rhizobium* inoculation affected AlkP. However, AcdP activity reached its maximum value with R1 treatment (15.5 ± 1.2 µg pNP g DM^−1^ h^−1^), while the minimum AcdP activity (10.2 ± 0.5 µg pNP g DM^−1^ h^−1^) was observed in the non-inoculated treatment (Fig. 1c). The highest DH activity was found in the R1 treatment (57.2 ± 7.4 µg TPF g DM^−1^ 24^−1^) that showed double the activity in comparison with the other treatments (Fig. 1a).

Contrarily to the effect observed for AcdP and DH, the highest UE activity (53.9 ± 6.9 µg NH_4_-N g DM^−1^ 2 h^−1^) was observed in the non-inoculated treatment, while the minimum UE activity (34.4 ± 3.7 µg NH_4_-N g DM^−1^ 2 h^−1^) was measured in R1 (Fig. 1e). β-Glu activity was the only soil enzyme activity significantly affected by both the application of essential oil and the inoculation with different *Rhizobium* strains (*p* < 0.001 for both factors). The maximum β-Glu activity (358 ± 29 µg PNP g DM^−1^ h^−1^) was observed in the non-inoculated treatment, while the lowest was found in R3 (188 ± 27 µg PNP g DM^−1^ h^−1^) (Fig. 1b). In line with this, the application of essential oils also induced a reduction of the β-Glu activity; higher values of this enzyme activity were observed in the E0 treatment than in E1 and E2.

### Influence of essential oil and Rhizobium inoculation on plant properties

The effects of the application of different *Rhizobium* inoculation treatments on plant height are displayed in (Table 5). Plant height slightly increased with the application of *Rhizobium* strains; however, for R1 and R2 in combination with the lower essential oil dose (E1), a significant decrease was observed (Tables 3 and 5). Contrarily, for R3 a strong increase in plant height of almost 10 cm was observed compared to the other treatments (non-inoculated, R1, R2). However, we found reduced growth with the lower essential oils dose (E1) in comparison to E0 and E2. As expected, the number of nodules measured was influenced by the different inoculation treatments (*p* < 0.001). A higher average number of nodules was observed for R2 and R3 (44.3 and 50.4, respectively for both treatments), compared to R1 (29.5 nodules) and the non-inoculated control, for which no visible nodules were observed. Moreover, significant interaction between both treatments (Inoculation × Essential oil; *p* < 0.05) was observed for the number of nodules (Tables 3 and 5) in the case of plants inoculated with R3, being the treatment R3 E1 (Table 3) the one presenting the highest nodule number. Although no significant effects of any of the applied treatments were observed for plant biomass (Table 3), it can be noticed that, on average, plants inoculated with *Rhizobium* strains (R1, R2, and R3) yielded a higher biomass than the control one (Table 5). Similarly, inoculation did not affect plant N-content (Table 5); however, plants grown in those soils inoculated with R1, R2 and R3 showed higher nitrogen contents (2.4, 2.2, and 2.7 g kg^−1^ respectively) than the non-inoculated control (approx. 1.8 g kg^−1^).

**Table 5:** Two-way analyses of variance (ANOVA) for plant parameters of *Phaseolus vulgaris L*. treated with rhizobial inocula and essential oil as the source of variance.

Parameters	Inoculation treatment		Essential oil treatment		Inoculation × Essential oil	
	*F*	*p*	*F*	*p*	*F*	*p*
Nodules	217.3	*p* < 0.001	n.s.	n.s.	3.18	*p* < 0.05
Plant height	9.7	*p* < 0.001	n.s.	n.s.	n.s.	n.s.
Total plant biomass	n.s.	n.s.	n.s.	n.s.	n.s.	n.s.
Plant N content	n.s.	n.s.	n.s.	n.s.	n.s.	n.s.

n.s.: Not significant.

## Discussion

The presence of plant growth promoting bacteria (PGPB) in agricultural soils and their dynamics with plants has gained increasing interest among the scientific community thanks to the abilities of PGPB to solubilize nutrients^42–44^. In fact, the application of Rhizobia and plant extracts in agriculture could be considered as a potential strategy for their application in organically managed and ecologically oriented farming systems. This is also true for the combined effect of *Achillea millefolium* essential oil and *Rhizobium* inoculation based on their potential influence on soil chemical, biological and plant growth parameters. Moreover, to our knowledge, there are no studies yet that have considered these factors.

For all of the combinations of *Rhizobium* with essential oil, a reduced abundance of both bacterial and fungal CFUs was measured in the present study. However, in the case of the R0E0 treatment, the bacterial CFUs were significantly higher than in the remaining treatments. Oils are known to have inhibitory effects against microbial populations and, in particular essential oil extracted from *Achillea millefolium L* has shown antimicrobial and antifungal properties^15^. Although the antifungal effect in the treatments containing oil was significant, such effect was not as strong as that registered for bacteria.

In our study, it has been observed that even a small amount of essential oil had a positive effect in combination with *Rhizobium* to suppress both the bacterial and the fungal abundances. However, the specific oil component responsible for this suppression, and the underlying mechanisms behind this effect are not clear yet.

In line with our results, where we observed that the inoculation with 2 of the 3 strains led to a reduction in bacterial abundance, Christensen and Jakobsen^45^ also recorded a decrease in the bacterial abundance in cucumber plants after four weeks following inoculation with vesicular-arbuscular mycorrhizal fungi (AMF). Also, a pronounced antibacterial response of oil extract from *Achillea millefolium* on *Staphylococcus* species was reported^46^. Moreover, the inhibitory effect of essential oils on several plant pathogens has been observed in previous studies^47–49^. However, other studies reported an enhanced soil metabolism and microbial activity following the application of essential oils^50–52^. In addition, it has been demonstrated that small amounts of *Lavandula stoechas L*. essential oil have a positive effect on mycorrhiza development^53^. Thus, the utilization of plants containing this essential oil in agriculture can be beneficial due to the natural origin of such oils, and their efficient action against pathogens based on the fact that they contain multiple active compounds, which hampers pathogen resistance development^54^.

Soil microbial activity, measured as basal respiration in this study, reached higher values when no essential oil was applied for R0 and R1; and decreased with the addition of essential oil except for the R3E2 treatment. Several researchers have documented an enhancement of soil respiration after the application of essential oils and their constituents^50,55^. A plausible explanation could be that the application of this type of oils induced a shift in the soil microbial community favouring the appearance of bacterial strains that are resistant and/or capable of catabolizing such components^51^. Assuming this, we would expect that some biologically active secondary metabolites derived from the extracted essential oil of *Achillea millefolium L* might be able to alter soil microbial communities and promote the beneficial microbes involved in a rapid colonization of rhizosphere. However, the short duration of our experiment (45 days) made it impossible to confirm such observations.

In this experiment, the inoculation with different *rhizobial* strains induced a decrease in most of the measured soil enzyme activities, in particular DH, which can be linked to the soil microbial biomass since its activity serves as an indicator for the physiologically active soil microbiota^56,57^. Indeed, some essential oils and the individual constituents do not cause an immediate effect on the soil microbiota, but rather seem to require a certain period of time, between 4 and 16 weeks depending on the studies, before increasing soil microbial abundance and basal respiration^50,55^. However, it seems that R1 bacteria are more resistant than R2 and R3 bacteria to the effect resulting from the application of *Achillea millefolium* essential oil. Contrary to AlkP, AcdP activity reached its maximum level following R1 inoculation, while the lowest AcdP activity was observed in the no inoculation treatment. Although alkaline phosphatase is an enzyme produced during microbe-plant interactions^58^, it showed a positive response to the application of all *Rhizobium* inoculation and essential oil combinations compared to the no inoculation treatment. Nevertheless, numerous studies have demonstrated that the activity of alkaline phosphatase can remain unchanged after several treatments like those resulting from silvicultural activities^59^.

DH activity showed a significant decline depending on the bacterial species. In addition, DH activity which is primarily involved in the carbon cycle has a pivotal role in the enzyme system of all microorganisms^57^ that are linked with soil microbial respiration. The abovementioned activity decrease occurred after both inoculation and essential oil treatments. Both treatments also induced a reduction in soil microbial abundance that could explain the decrease of β-Glu activity, which seems to be highly related to the reduction in microbial carbon^60^. As UE activity was strongly affected by the inoculation treatments used in our study, the decrease in this enzyme activity could be due to nitrogen utilization by bacteria and reduction in soil N available forms^61^. In addition^62^, reported that the amendment with different natural products may act as an inhibitor of UE activity.

In contrast to our expectations, *Rhizobium* inoculation and the treatment with essential oil doses neither affected NH_4_^+^ N, NO_3_^−^ N nor total N contents in soils, despite the increased number of root nodules in the inoculated treatments. However, since in the present study the whole plant was collected, the additional N accumulated in nodules was not added to the soil because it is released only during the decomposition of legume biomass. The rotating cropping system considers the release of N *in situ* either of the whole biomass, or of the roots and nodules^63^. Another plausible explanation for the non-significant effect could be related to the short duration of the experiment. Nevertheless, it is important to highlight that plants amended with both treatments showed similar NH_4_^+^ N, NO_3_^−^ N, and total N contents than those from the treatments with no essential oil doses. This indicates that the application of these extracts affects soil microbiota in the short term rather than soil nutrient contents.

We observed that *Rhizobium* inoculation in combination with all of the rates of essential oil showed a positive response in terms of nodule number and plant height. Nevertheless, inoculating common bean and amending with essential oil did not result in a significant increase in plant biomass and plant N content. Previous studies have reported that *Rhizobia* are considered a main agent for N fixation in plants, root nodulation and crop growth^64–66^. Boddey *et al*.^67^ found that inoculation of Cowpea with the *Rhizobium* strain BR 3299 significantly increased grain yield. Furthermore, as stated in Fan *et al*.^68^, inoculation of tomato plants with Plant Growth Promoting Rhizobacteria enhanced N and P uptake in the shoots. Contrary to these authors, and in line with our observations, Chekanai *et al*.^69^ observed that inoculation with *Rhizobium* did not induce a positive response in plant biomass or grain yield in common bean (*Phaseolus vulgaris L*). However, it is known that essential oils are proven to be a thousand times less toxic than standard insecticides, and many of their constituents are not persistent in freshwater and can be metabolized by soil-borne microorganisms^10^.

## Conclusions

In summary, we conclude that the application of *Achillea millefolium* essential oils alone or in combination with *Rhizobium* bacteria had an influence on soil productivity and the development of common bean (*Phaseolus vulgaris* L.). The biocide effect of the application of these essential oils was reflected in a decrease in bacterial and fungal CFUs as well as in basal respiration and soil enzymes activities. Moreover, such effects were stronger with higher oil concentrations. However, the applied essential oil did not seem to harm the effectivity of the used *Rhizobium* strains, in fact, its joint application increased the number of nodules and the plant height in the studied bean plants. Such effects were not accompanied by an increase in neither inorganic nor total N content in soil, which could be due to the duration of the study. In order to corroborate the positive results of this study, it would be necessary to perform further long-term studies under field conditions to determine the upscale application of *Achillea millefolium* essential oils, and their potential as additives suitable for organic farming and biologically managed soils.
